# Successful Non-fluoroscopic Radiofrequency Ablation of Incessant Atrial Tachycardia in a High Risk Twin Pregnancy

**DOI:** 10.1016/s0972-6292(16)30712-4

**Published:** 2014-01-01

**Authors:** Zia Zuberi, John Silberbauer, Francis Murgatroyd

**Affiliations:** Department of Cardiology, King College Hospital, Denmark Hill, London

**Keywords:** Ectopic atrial tachycardia, Tachycardia-cardiomyopathy, Twin pregnancy

## Abstract

We describe a patient presenting with incessant ectopic atrial tachycardia during a high risk twin pregnancy. Tachycardia was resistant to escalating doses of beta-blockade with digoxin. Because of increasing left ventricular dysfunction early in the third trimester, catheter ablation was performed successfully at 30 weeks gestation. Electro-anatomic mapping permitted the entire procedure to be conducted without the use of ionizing radiation. The pregnancy proceeded to successful delivery near term and after three years the patient remains recurrence free with normal left ventricular function, off all medication.

## Introduction

Arrhythmias may be facilitated in pregnancy by altered autonomic tone, neuro-hormonal effects and cardiac chamber stretch secondary to increased intravascular volume. They are usually self-limiting, and seldom pose a risk to maternal or fetal health. Treatment is generally conservative though pharmacological therapy is sometimes indicated on symptomatic and occasionally prognostic grounds. Incessant (usually modest) tachycardia may be misdiagnosed particularly during pregnancy when sinus tachycardia is common. Failure to recognise and treat incessant tachycardia may result in cardiomyopathy which in pregnancy is associated with poor fetal and maternal outcome. Ablation of drug-resistant arrhythmia has previously been described in singleton pregnancy. Non-fluoroscopic navigation systems have facilitated both the mapping of complex arrhythmias and ablations, and the reduction of exposure to ionising radiation. There are however no previous reports of catheter ablation performed in twin pregnancy.

## Case

**Figure 1:**
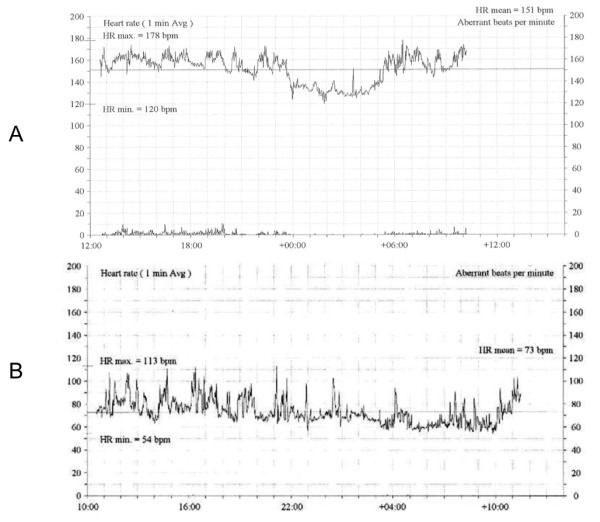
Mean heart rate tachograms recorded over a 24hr period pre (A) and post ablation (B).

A 48 year old with multiple previous miscarriages secondary to thrombophilia and no previous successful pregnancy became pregnant with twins after repeated in-vitro fertilisation (IVF) cycles. The patient was noted to have an asymptomatic tachycardia and initially started on labetolol 100mg b.i.d. She was referred for assessment to the arrhythmia service. At 28 weeks' gestation mean heart rate was 151 beats per minute (bpm) (Figure 1A) but the patient remained asymptomatic.

**Figure 2:**
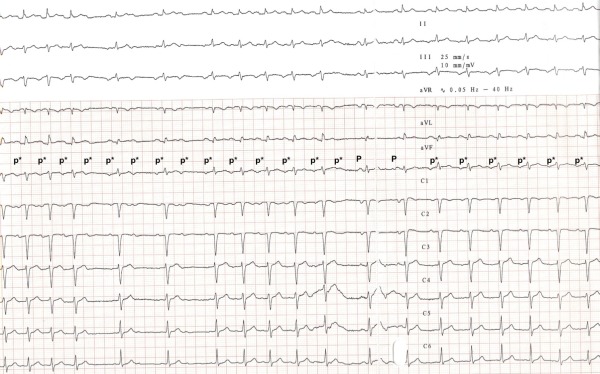
12-lead ECG demonstrating tachycardia. During recording an adenosine bolus was given which transiently induces AV block without alteration of atrial cycle length. Cardioversion to sinus rhythm is seen for 2 beats before resumption of tachycardia. Subtle morphological difference can be seen between tachycardia (p*) and sinus (P) beats. Tachycardia p-waves are noted to be negative in V1 and positive in aVL consistent with a right atrial tachycardia focus.

A 6mg adenosine challenge with 12-lead ECG recording was performed (Figure 2). Adenosine transiently induced atrioventricular block followed by two sinus beats. This permitted characterisation of P-wave morphology (positive in aVL, negative in both V1 and inferior leads. P-waves were isoelectric or positive in the precordial leads) which was suggestive of a tricuspid annular or low right atrial focus.

Immediate resumption of the tachycardia indicated that cardioversion would not be clinically useful. Beta-blockade was escalated and digoxin added, but ventricular rate control remained poor and left ventricular function was clearly deteriorating. At 30 weeks' gestation, the mean ventricular rate was 126 bpm despite labetalol 300mg t.i.d. and digoxin 250mcg daily, and the left ventricular ejection fraction was 30%, indicating tachycardia-cardiomyopathy early in the third trimester, when hemodynamic demands increase greatly. Treatment options were discussed (early delivery by Caesarean section, further medical therapy or catheter ablation). Owing to anticipated further deterioration in LV function with progression of pregnancy the patient elected for catheter mapping and ablation at 30 weeks' gestation.

**Figure 3:**
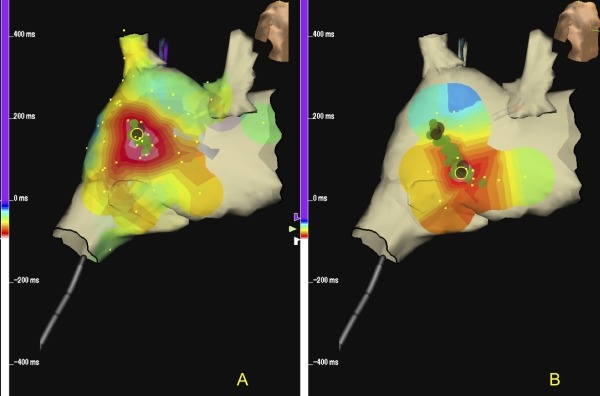
3D activation map confirming focal tachycardia mechanism with earliest activation lateral to the mid crista terminalis (A) with centrifugal spread from this point. Ablation lesions at this site modified tachycardia to a slower tachycardia which was mapped and ablated to sinus rhythm just lateral to the low crista (B).

Venous access was gained via the right femoral vein under local anaesthesia. Owing to hemodynamic instability when supine due to IVC compression by the gravid uterus, the rest of the case was conducted with the patient in the left semi-decubitus position. Multipolar diagnostic and mapping catheters were advanced without fluoroscopy to the right atrium (Halo ®, Biosense Webster, Diamond Bar, CA and 4mm Blazer XP ® Boston Scientific, Natick MA) and coronary sinus (Livewire ® St Jude Medical, Minnetonka MN). The Halo catheter was positioned in the right atrium and rotated in multiple planes to facilitate high resolution data point geometry collection from the 20 electrodes of the Halo catheter with final positioning in a circumferential manner around the right atrium with some poles positioned posteriorly along the crista terminalis. Intracardiac electrograms demonstrated an atrial tachycardia cycle with a mean cycle length of 525ms, and proximal to distal activation of the coronary sinus. Electroanatomic mapping (EnSite NavX®, St Jude Medical) was used to guide catheters towards and within the heart, and to create an activation map of the tachycardia. This confirmed a focal mechanism with earliest activation adjacent to the mid crista terminalis (Figure 3A).

**Figure 4:**
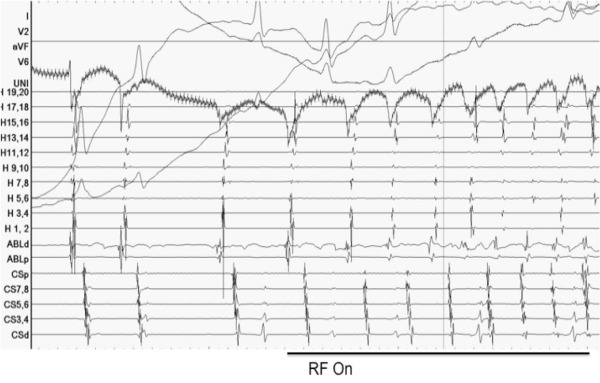
Intracardiac signals. Displayed are surface ECG, Unipolar EGM, Halo-catheter EGM (H1-H20), ablation catheter and coronary sinus EGM (CS 1-10). The Halo catheter was positioned circumferentially (roof (H15-20)>lateral wall (H5-14)>floor (H1-4)) around the right atrium with some poles straddling the crista terminalis (double potentials). The recording demonstrates a QS early signal on unipolar mapping together with tachycardia acceleration at the onset of RF ablation.

The local electrogram activation preceded surface P-wave by 20ms with a sharp negative deflection on unipolar electrogram (Figure 4). Radiofrequency ablation just lateral to mid crista resulted in slowing of tachycardia to a modified tachycardia cycle length of 620ms. Remapping indicated earliest activation from a more inferior exit along the lower crista (Figure 3B, possibly a second breakout from a modified focus), where further ablation terminated tachycardia. The patient remained in sinus rhythm thereafter, with a significant reduction in mean heart rate (Figure 1B) associated with clinical improvement in LV ejection fraction (EF 39%). Elective delivery by Caesarean section at 37 weeks resulted in successful delivery of healthy twins. At three years' follow-up there has been no arrhythmia recurrence and LV function remains normal (LVEF 53%), despite withdrawal of all medical therapy.

## Discussion

Incessant tachycardia in pregnancy can result in adverse maternal and fetal outcomes, including prematurity, intrauterine growth retardation, respiratory distress and congenital heart disease [1]. Persistent tachycardia may be asymptomatic while still giving rise to maternal cardiomyopathy. Antiarrhythmic drug therapy to treat maternal tachycardia is potentially harmful to the fetus particularly in the first trimester owing to the risk of teratogenicity. Drugs such as digoxin, beta blockers and flecanide are generally considered safe later in pregnancy but drug metabolism and drug bioavailability issues can limit efficacy. Amiodarone is associated with fetal hypothyroidism, intrauterine growth retardation, fetal bradycardia and QT prolongation. In this case, rate control with drug therapy failed, leading to LV dysfunction (precluding most antiarrhythmic drugs), and potential fetal compromise. Early in the third trimester deteriorating LV function prompted the choice of attempted catheter ablation rather than risking sudden hemodynamic decompensation or fetal distress.

Ionizing radiation is potentially harmful to the fetus even in late pregnancy [2] and exposure should be minimised by correct shielding, low frame rates, and short fluoroscopy times. Modern electroanatomic mapping systems permit both placement of catheters within the heart and characterisation of tachycardia with minimal fluoroscopy, and in this case, successful mapping and catheter ablation was performed entirely without exposure to X-Rays [3,4].

There are few previous reports of catheter ablation in pregnancy. A series from four centres described successful catheter ablation of various supraventricular tachycardias SVT in nine singleton pregnancies (four mothers had impaired left ventricular function). There were no complications for mother or fetus [1], and the mean fluoroscopy time was only 42±37s. Ferguson et al. performed a left atrial focal tachycardia ablation without fluoroscopy in pregnancy with electroanatomic mapping and intracardiac echo to facilitate transeptal puncture [5]. These reports highlight the potential benefits of catheter ablation if drug therapy proves unsuccessful and is consistent with American Heart Association (AHA) guidelines that have recommended consideration of catheter ablation with minimal fluoroscopy in the second trimester if drug therapy fails.

There have been no previous reports of successful radiofrequency ablation in twin pregnancy. The decision to perform ablation in this case was not straightforward. A continued conservative approach had a high risk of adverse outcome to mother and fetus, because of maternal age, twin pregnancy, multiple previous miscarriages and established tachy-cardiomyopathy. Cardioversion was not performed as the patient's tachycardia was incessant re-initiating immediately after termination as is often the case with such arrhythmias (Figure 2). The risk benefit ratio for a right-sided procedure was therefore felt to be in favour of attempted catheter ablation.

Twin pregnancy can result in specific problems such as catheter advancement from femoral access sites owing to external compression of the vena cava from the gravid uterus. The gravid uterus may also cause significant hemodynamic instability secondary to external IVC compression syndrome effecting venous return to the heart. In this case after establishing venous access the patient was repositioned in the left semi-decubitus position to facilitate venous return. This did not affect mapping as 3D mapping allows rotation of the created geometry.

Based on previously reported successful ablation in singleton pregnancy we report extension of these observations to a high-risk twin pregnancy. In this case fluoroscopy was entirely avoided using electroanatomic mapping, though brief exposure may be needed if difficulty is encountered with catheters in the great veins or for safe trans-septal puncture.
